# 

**Published:** 1898-11-26

**Authors:** 


**Notices of Births, Deaths, and Marriages are inserted in this column at a charge of 2s. 6d. for an announcement not exceeding four lines.**

### NOTICE TO CORRESPONDENTS.

All MS., Letters, Books for Review, and other matters intended for the Editor should be addressed The Editor, "The Hospital," The Hospital Building, 28 & 29, Southampton Street, Strand, W.C.

The Editor cannot undertake to return rejected MS., even when accompanied by stamped directed envelope.

**Notice to Advertisers and Subscribers.**

All Advertisements, Orders for Copies of The Hospital, and Business Communications should be addressed to **THE MANAGER (Not to the Editor),** The Hospital, The Hospital Building, 28 & 29, Southampton Street, Strand, London, W.C.

The Cost of Subscription, post free, is as follows:—Per week, 2½d.; per quarter, 2s. 8d.; per half-year, 5s. 3d.; per annum, 10s. 6d. Foreign:—Per annum, 15s. 2d., payable, in all cases, in advance.

**NOTICE.—Vol. XXIV. of "The Hospital" (April, 1898, to September, 1898), in handsome cloth binding, is now on sale at the Office of this Journal, price 7s. 6d. Cases for binding the Numbers contained in this Volume 1s. 6d. Index to Vol. XXIV., 2½d., post free.**

**The Monthly Part for December will be ready on December 1st. Price 6d., by post 8d.**

### BOOKS RECEIVED.

WEST, NEWMAN, AND CO.

"Archives of Surgery." By Jonathan Hutchinson, LL.D., F.R.S.

H. K. LEWIS.
"Drs. Harvey and Davidson's Syllabus of Materia Medica." By William Martindale, F.L.S., F.C.S.

C. ARTHUR PEARSON.

"Strange Stories of the Hospitals." By Frank Aubrey.

BAILLIERE FILS.

"La Pratique des Maladies des Enfant dans les Hopitaux de Paris." Par le Professeur Paul Lefert.

THE SCIENTIFIC PRESS.
"Some Health Aspects of Education." By Dr. Percy Lewis.

CASSELL AND CO.

"Gout: Its Pathology and Treatment." By Arthur P. Luff, M.D. Lond., F.R.C.P.

METHUEN AND CO.
"To Arms." By Andrew Balfour. Illustrated by Cecil W. Quinnell.

### SCRAPS AND GLEANINGS.

Pound Day at the Southampton Free Eye and Ear Hospital was very successful financially. About £40 has been received in cash, as well as the numerous pounds and half-pounds of tea, &c.

A complete Röntgen rays apparatus has been presented to the North-Eastern Hospital for Children, Hackney Road, Shoreditch, by the Duke of Newcastle, who is a member of the committee. The apparatus, which is by Mr. J. Le Couteur, throws an 18 in. spark, and is one of the largest and most efficient in existence.

An earnest appeal is being made to build a permanent home for the Littlestone-on-Sea Convalescent Home. It is proposed that the new home shall contain about 75 beds, and one ward specially adapted to the requirements of mothers with their infants. The cost will not be less than £5,000, towards which upwards of £1,600 has been collected.

The Bethnal Green Library appears to be doing very useful work. Apart from providing large, airy, and well-lighted rooms wherein those who want to read may enjoy their reading, they have instituted evening classes where a variety of subjects are taught, and there are also evening lectures held in the Great Assembly Hall, which are much appreciated.

Mr. Alderman Treloar is again working with a view to distributing Christmas hampers to the whole number of deserving crippled children (upwards of 5,000) registered on the books of the Ragged School Union. Contributions addressed to Mr. Alderman Treloar will be gladly received at 68, Ludgate Hill. Last year hampers were distributed to 4,000 children.

The report of the sub-committee appointed to consider the question of abuse at the Coventry and Warwickshire Hospital has been accepted. One of the recommendations of the sub-committee is to the effect that on and after November 1st every patient shall be required to present a ticket before receiving treatment, excepting in the case of simple teeth extractions for children under twelve, accidents, and such acute medical cases as require immediate help.

